# The first case of human autochtonous subconjunctival dirofilariosis in Poland and MALT lymphoma as possible consequence of this parasitosis

**DOI:** 10.1186/1750-9378-10-1

**Published:** 2015-01-07

**Authors:** Piotr K Borkowski, Grzegorz Rymkiewicz, Joanna Golebiewska, Nestor Nestoros, Joanna Romejko-Jarosinska, Hanna Zarnowska-Prymek, Aleksander Masny, Jakub Palucki, Danuta Cielecka

**Affiliations:** Department of Zoonoses and Tropical Diseases, Medical University of Warsaw, Warsaw, Poland; Flow Cytometry Laboratory, Department of Pathology and Laboratory Diagnostics, The Maria Skłodowska-Curie Memorial Cancer Center and Institute of Oncology, Warsaw, Poland; Department of Ophthalmology, Medical University of Warsaw, Warsaw, Poland; Department of Lymphoid Malignancies, The Maria Skłodowska-Curie Memorial Cancer Center and Institute of Oncology, Warsaw, Poland; Department of Medical Parasitology, National Institute of Public Health – National Institute of Hygiene, Warsaw, Poland; Department of Radiology, The Maria Skłodowska – Curie Memorial Cancer Center and Institute of Oncology, Warsaw, Poland; Department of General Biology and Parasitology, Medical University of Warsaw, Warsaw, Poland

**Keywords:** *Dirofilaria repens*, Human, Poland, Autochtonous species, MALT lymphoma, *Wolbachia*

## Abstract

The first case of human dirofilarosis in Poland was recorded in 2007. Until that time our country was free of *Dirofilaria repens.* Recent studies show that 21,4- 60% of dogs in Warsaw region harbour microfilariae, therefore it is becoming a growing problem in Central Europe.

In April 2013 a subconjunctival *D. repens* was removed from the eye of 61-year-old woman. It was the twenty first case of this disease in Poland, the third case of eye dirofilaria and the fourth autochtonous case. The patient had never been abroad, so it was the first case of autochtonous human ocular dirofilariosis in Poland. Nine months after the *D. repens* had been removed, a MALT lymphoma was discovered. In the article we discuss whether a MALT lymphoma of the lacrimal gland of the eye, previously affected by the parasite, may be the consequence of the invasion.

## Introduction

Dirofilariosis is a parasitic infection diagnosed mainly in dogs and less frequently in cats or free living carnivores. Occasionally dirofilariosis can be the zoonotic human infestation. However, this happens rarely, when *Culex*, *Aedes* or *Anopheles* mosquitoes, during blood consumption, inject microfilariae into human circulation. In Poland, *Dirofilaria (Nochtiella) repens* (Railiet et Henry, 1911) is responsible for the disease in the form of subcutaneous nodules and of subconjunctival localization [1–3]. The first cases of dirofilarosis in Poland was recorded in 2007 [2, 4]. Until then, Poland and other neighboring Central European countries were free of *D. repens.* Initially, we suspected that all of the cases of human dirofilariosis were brought from abroad. Recent studies have showed that 21,4% up to 60% (average 25,8%) of dogs residing in Warsaw area harbour microfilariae. Such percentage is almost equal to the one in Italy, a traditional endemic region, therefore atochtonous dirofilariosis it is a growing problem in Poland and Central Europe [4–7].

Below the case of lacrimal gland MALT lymphoma which was discovered nine months after removing of *D. repens* from conjunctiva of the affected eye is presented.

## Case report

**Figure 1:**
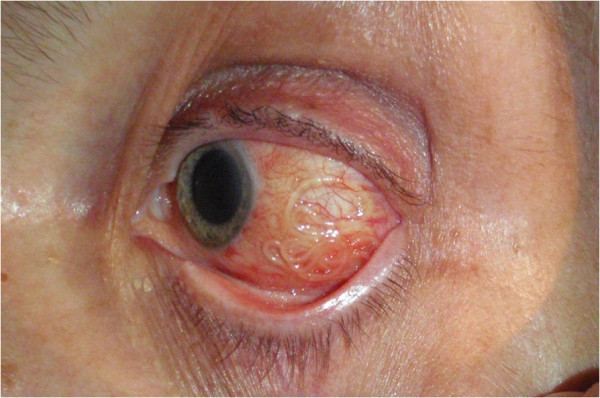
**Eye.**

**Figure 2:**
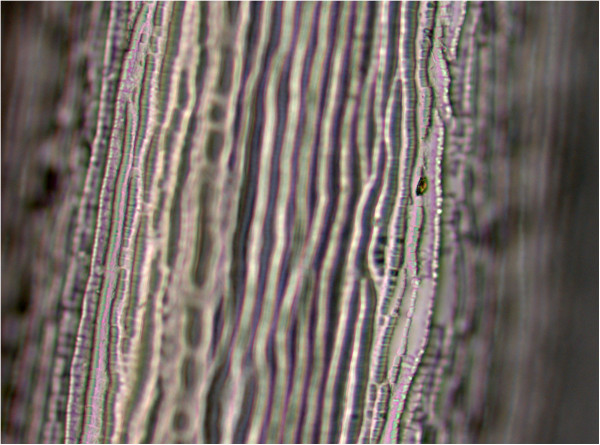
**Stripes and ridges.**

In July 2012, a 61-year-old woman came to the Ophthalmology Department of Warsaw Medical University with the history of one day pain and itching in her left eye. A subconjunctival worm was well-visible (Figure 1). An ophthalmologist removed the worm surgically and referred the patient to the Zoonoses and Tropical Diseases Department for further investigation. It was 11 cm long and 0,47 mm wide nematode. Microscopic investigation proved that it was a young female with uterus filled with oocytes. Characteristic longitudinal ridges and delicate transverse stripes typical for *D. repens* cuticle were recognized (Figure 2).

Dirofilaria DNA was amplified using DR COI-F1 and DR COI-R1 primers using real-time PCR. Positive results of the amplification of the gene of the first subunit of cytochrome oxidase and the analysis of melting curves of PCR products confirmed that our nematode belonged to the species of *D. repens* [8].

Microfilariae in patient’s peripheral blood were not detected. ELISA test for human tropical filariosis (Bordier, Switzerland, which use *Acanthocheilonema vitae* somatic antigens) and for *Toxocara canis* were negative. There was no eosinofilia. Before obtaining all these results, in case of microfilariae presence, we undertook the albendazole treatment [9]. A few days after the surgery, the patient’s eye was healed, although occasionally she reported a delicate pain. The patient’s dog was also examined for presence of microfilariae, but the results were negative.

**Figure 3:**
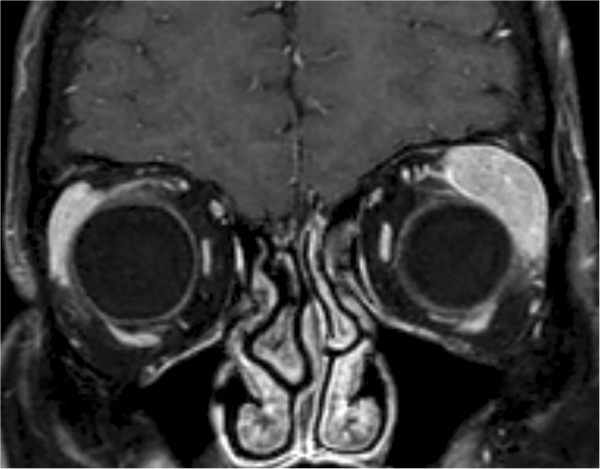
**MRI of orbital tumor.**

**Figure 4:**
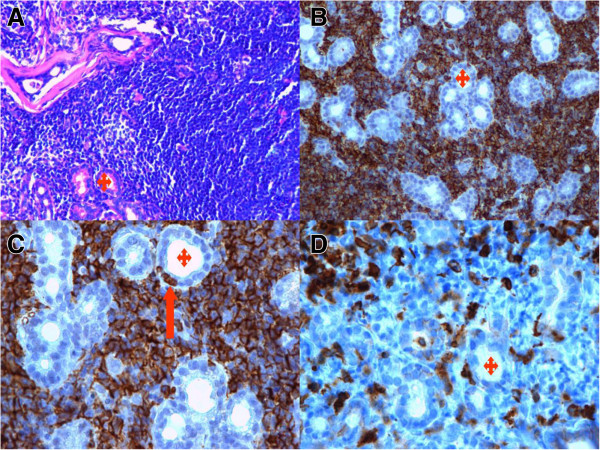
**Morphological and immunohistochemical features of extranodal marginal zone lymphoma of mucosa-associated lymphoid tissue (MALT lymphoma) of ocular adnexal. A)** Monotonous population of small centrocytic cells with scant cytoplasm (ocular adnexal section HE, ×200 magnification), **B)** MALT lymphoma cells express CD20 (+).There are scattered glands of ocular adnexal (red stars in all figures) (CD20 immunostain ×200 magnification), **C)** MALT lymphoma with few lymphoepithelial lesions - the tumour cells infiltrate the glands (red arrow) (CD20 immunostain ×400 magnification), **D)** MALT lymphoma with low number of normal T CD3 (+) lymphocytes (CD3 immunostain ×400 magnification). In addition to the figures, the lymphoma cells are negative for CD43 (−) and CD5 (−). Plasmacytic differentiation is present in approximately 20% of MALT cells with CD138 (+) expression on this subpopulations of lymphoma cells. Tumour cells show kappa (+)/lambda (−) light chain restriction by immunohistochemical reaction.

In April 2013 the patient returned to the Zoonoses and Tropical Diseases Department with the pain in the same eye. Despite conjunctivitis we did not find any abnormalities, so she was referred to the Ophthalmology Department. Within one week she developed periorbital edema and diplopia. The ultrasound and MRI revealed an enlarged lacrimal gland (Figure 3). Tumor samples were taken for histopatological examination [10]. The diagnosis was extranodal marginal zone lymphoma of mucosa associated tissue (MALT lymphoma) (Figure 4). The patient was referred to the Institute of Oncology for further treatment. Until present (July 2014), she undertook the six R-COP (Rituximab, Cyclophosfamide, Vincristine, Prednisone) immunochemotherapy courses and her status remains excellent.

## Discussion

The patient resides near Warsaw and she has never been abroad, so the case is autochtonous. It was the twenty first case of dirofilariosis, the third case of the eye dirofilaria and the fourth autochtonous case in Poland, but the first ocular and autochtonous. The first case of human dirofilarosis in Poland was recorded in 2007. Until then, Poland and neighboring Central European countries were free of *D. repens*. At first, we considered all human cases to be transferred from abroad, but currently we know that many of them are autochtonous [1]. The first cases of animal and human dirofilariosis in Poland were described only in Warsaw surroundings (Mazowieckie Province). Later studies detected microfilariae in dogs blood of all regions, but the largest percentage is still in Mazowieckie Province- 25,8% average [5]. This region is the richest in Poland, so people from here travel most frequently. Similar percentage of dogs are affected in traditional endemic regions in Italy even temperatures in Poland are lower. Autochtonous dirofilariosis is becoming a growing problem in Poland. The situation described is the result of global warming or it might be also the result of the fact that just in 2007 Poland had joined the Schengen group (EEC no-borders countries). Since then the number of people travelling with their dogs to the endemic regions in Mediterranean region have grown extensively [11, 12].

Parasites most frequently live in subcutaneous tissue of the human body. Italian authors estimate that in about 30% of cases worms are situated in ocular region, whereas similar literature from Ukraine estimate that up to 40% of cases are located there [7, 13]. In this localization infection may be periorbital, subconjunctival or most rarely intraocular [10, 14]. In literature we found two cases where worms were removed from lacrimal gland: one was in China and another one in Croatia [15, 16].

Microfilarial migration lasts months before reaching maturity and adult parasite can persist 2–4 years in human body. Granulomatous inflammation around parasite forms usually a tender nodule. It produces local irritation, edema, sometimes itching. In literature it was described several times, that these nodules were initially taken under the consideration as a malignancy, and only later histological assessment elucidate their true origin [17]. This patient did not feel any under-the-skin movement so the worm grew behind her eye ball, possibly in the lacrimal gland. There was no eosinofilia, itching nor pain and the serological tests were negative so in this case inflammation was not intense but long-lasting.

There are a number of situations, where parasites (from the trematoda) are responsible for carcinogenesis: eggs of *Schistosoma haematobium* in urinary bladder wall and eggs of *S. japonicum* in a large intestine wall are responsible for squamous cell carcinomas, *Opisthorchis* and *Clonorchis* can cause cholangiocarcinoma [18]. Until recently there was no data of cause and effect linkage between helminths and malignant transformation in the orbit area. Tumor was discovered (became clinically significant) nine months after the worm was removed, but we can’t exclude, that the transformation occurred during parasite presence.

On the other hand, in many cases of MALT lymphoma, there is a history of a chronic inflammatory disorders that results in accumulation of extranodal lymphoid tissue. The chronic inflammation may be the result of infection, autoimmunity or other unknown stimuli. Examples of infectious organisms that may cause accumulation of mucosa associated tissue preceding MALT lymphoma include: *Helicobacter pylori* (gastric MALT lymphoma), *Chlamydia psittaci* (ocular adnexal MALT lymphoma) and *Borrelia burgdorferi* (cutaneus MALT lymphoma) [19–22]. At least in ocular MALT lymphomas, there is great variation in the strength of these associations that might relate in part to geographic diversity [23]. There is a documented coexistence of bacterial endosymbiont *Wolbachia* with *D. repens* [24, 25]. Does *Wolbachia*, as an unknown stimulus, play a role in pathogenesis of MALT lymphoma in this case?

## Conclusions

1. The number of human cases of dirofilariosis in Poland and Central Europe will increase in the future approaching the level similar in the traditional endemic regions. This subsequent autochtonus case supports this hypothesis.
2. It has been proved that increasing cases of dirofilariosis in Central Europe resulted from global warming, but it is worth to note that it might also be the result of increased traveling of people with their dogs after Poland and neighboring countries have joined the Schengen group in 2007.
3. We postulate that *Dirofilaria repens* invasion and/or *Wolbachia* could play a role in pathogenesis of ocular MALT lymphoma as possible factor.

### Consent

Written informed consent was obtained from the patient for publication of this report and any accompanying images.
